# Neoadjuvant chemoradiotherapy with capecitabine based regimen in locally advanced rectal cancer: A retrospective study

**DOI:** 10.1097/MD.0000000000034985

**Published:** 2023-08-25

**Authors:** Fei Li, Chi Zhang, Liping Xu, Sheng Zhang, Dongsheng Zhang, Yan Leng, Chenjiang Wu, Jiayan Chen, Xinchen Sun

**Affiliations:** a Department of Radiation Oncology, The First Affiliated Hospital of Nanjing Medical University, Nanjing, China; b Department of Colorectal Surgery, The First Affiliated Hospital of Nanjing Medical University, Nanjing, China; c Department of Pathology, The First Affiliated Hospital of Nanjing Medical University, Nanjing, China; d Department of Radiology, The First Affiliated Hospital of Nanjing Medical University, Nanjing, China; e Department of Radiation Oncology, Fudan University Shanghai Cancer Center, Shanghai, China.

**Keywords:** DFS, locally advanced rectal cancer, neoadjuvant chemoradiotherapy (nCRT), OS, pCR

## Abstract

Capecitabine-based neoadjuvant chemoradiotherapy (nCRT) is the standard treatment for locally advanced rectal cancer. The objective of this study is to analyze overall survival (OS), disease-free survival (DFS) and prognostic factors of patients with stage II to III rectal cancer treated with nCRT in our institution. Between March 2014 to June 2020, 121 locally advanced rectal cancer patients were retrospectively reviewed and analyzed. All of the enrolled patients were treated with capecitabine-based nCRT (pelvic radiotherapy: 45–50.4 Gy, 1.8 Gy/d plus concomitant capecitabine-based chemotherapy), total mesorectal excision surgery (surgery was carried out 8–12 weeks after the end of CRT), and capecitabine-based adjuvant chemotherapy. We examined the pathological complete response rate, 3-year OS, 3-year DFS and the other prognostic factors. Kaplan–Meier method and Log-rank test were used to estimate and compare survival rate. With a median follow-up of 36 months, 3-year DFS and 3-year OS was 74.4% and 83.2%, respectively. Among the 121 patients, 24 achieved pathological complete remission (19.8%). After multivariate analysis, ypTNM stage (TNM stage after neoadjuvant therapy) was significantly associated with DFS. Positive mesorectal fasciae (MRF) status on magnetic resonance imaging and ypTNM stage were significantly related to OS. CRT with capecitabine based regimen provides high rates of survival and sphincter preservation with acceptable toxicity. YpTNM stage was significantly associated with DFS; magnetic resonance imaging MRF status and ypTNM stage were significant factors for OS after multivariate analysis. Distant metastasis is the dominant mode of treatment failure, and it is crucial to optimize systemic treatment for newly diagnosed patients.

## 1. Introduction

Neoadjuvant chemoradiotherapy (nCRT) followed by total mesorectal excision (TME) have become the standard treatment strategies for locally advanced (T3-4 or N+) rectal cancer (LARC) in the United States and Europe after several clinical trials.^[1–5]^ NCRT has led to a better quality of life (QOL) by increasing sphincter preservation rate, a decreased risk of local recurrence and a remarkably higher rate of pathologic complete response (pCR),^[6–8]^ and capecitabine-based CRT has become the standard regimen.^[9,10]^ PCR rates after neoadjuvant therapy ranged from 10.9% to 32%.^[11–16]^ The 5-year disease-free survival (DFS) was 68%, and the 10-year overall survival (OS) was 59.6% for the neoadjuvant chemoradiotherapy group of the German CAO/ARO/AIO-94 Randomized Phase III Trial.^[1]^

Recently, more attention has been given to the total neoadjuvant treatment (TNT)^[12,17–19]^ of LARC and the management of patients with clinical complete response (cCR) by the watch-and-wait strategy after nCRT for rectal cancer,^[20]^ but long-term outcomes need to be followed. Studies confirmed that TNT, compared to concurrent chemoradiotherapy (CRT) with adjuvant chemotherapy, can achieve a higher rate of Pathological Complete Response (PCR), and patients with lower rectal cancer may have a greater chance of sphincter preservation.^[19,21]^

In the present study, we describe the clinical outcomes of LARC patients who were treated in our institution with nCRT followed by TME and adjuvant chemotherapy. The purpose of this study is to analyze OS, DFS, and prognostic factors of patients with stage II-III rectal cancer treated with nCRT in our institution.

## 2. Materials and methods

### 2.1. Eligibility of patients

Patients aged 18 to 75-year-old with histopathologically confirmed as adenocarcinoma and lied ≤ 12 cm above the anal verge between March 2014 to June 2020. All patients were staged as locally advanced stage by contrast-enhanced magnetic resonance imaging (MRI), and metastases were excluded by chest and abdominal CT. According to the protocol, patients with one or more the following potentially high-risk factors were recommended to receive nCRT: a positive mesorectal fasciae (MRF) status on MRI; ≥cT3 tumor within 5 cm from the anal verge; a cT4 stage tumor that has adjacent organ involvement; or cN2 disease (≥4 positive lymph nodes). All patients’ physical condition and organ function were assessed to confirm that they were eligible for nCRT. Patients were also required to have a Karnofsky performance status score ≥ 70 and adequate marrow, liver, and renal function. The main exclusion criteria were confirmed metastatic disease, clinically significant cardio-cerebrovascular disease, and diagnosed peripheral neuropathy. The study was approved by the ethics committee of the First Affiliated Hospital of Nanjing Medical University (Nanjing, China). All patients provided written informed consent before the initial treatment.

### 2.2. Treatment

Radiotherapy was delivered with 6-MV photons via intensity-modulated radiation therapy (IMRT) or volumetric modulated arc therapy (VMAT) technique at a dose of 45 to 50.4 Gy in 25 to 28 fractions over 5 to 6 weeks. The clinical target volume (CTV) includes the primary rectum tumor, and any significant surrounding lymphadenopathy and high-risk nodal area, including the mesorectal, presacral, internal iliac and obturator lymph nodes. The planning target volume (PTV) was defined as the CTV plus a 0.7-cm margin to make up setup error. Patients received concurrent chemotherapy, either with capecitabine (825 mg/m^2^ twice daily 5 d/wk) or CapeOX (oxaliplatin 50 mg/m^2^ IV weekly and capecitabine 625 mg/m^2^ twice daily 5d/wk) during radiation therapy followed by capecitabine (1250 mg/m^2^ twice daily on days 1–14) or capecitabine combined with oxaliplatin (CapeOX) 2 weeks after the end of CRT (oxaliplatin 130 mg/m^2^ on day 1 and capecitabine 1000 mg/m^2^ twice daily on days 1–14) before surgery. Irinotecan in combination with capecitabine was used selectively in some patients based on the UGT1A1 status according to a randomized clinical trial^[15]^ (ClinicalTrials.gov identifier: NCT02605265). Capecitabine was administrated at 625 mg/m^2^ twice daily for 5d/wk, and weekly irinotecan (irinotecan was given weekly at a dose of 80 mg/m^2^ or 65 mg/m^2^ to patients with the UGT1A1*1*1 genotype or the UGT1A1*1*28 genotype, respectively), followed by 1 cycle of capecitabine plus irinotecan (XELIRI) 2 weeks after the completion of nCRT (irinotecan 200 mg/m^2^ on day 1 and capecitabine 1000 mg/m^2^ twice daily on days 1–14).

One study has shown that adding additional chemotherapy between the end of nCRT and TME surgery improved survival outcomes.^[12]^ We performed MRI examinations approximately 6 to 7 weeks after the completion of chemoradiation to evaluate the status of tumor regression. Then, 0 to 4 cycles of chemotherapy were arranged preoperatively after nCRT according to the patient’s condition and MRI assessment outcome. Generally, radical surgery was carried out 8 to 12 weeks after the completion of nCRT. Patients with poor tumor regression on MRI were considered for additional more chemotherapy courses.

We took patient’s age, comorbidities, the Karnofsky performance status score, surgical complications, and pathological stage into consideration before selecting adjuvant chemotherapy regimens. Adjuvant chemotherapy started 4 weeks after surgery, or when patients recovered from surgery. Treatment options included capecitabine (1250 mg/m^2^ twice daily on days 1–14) or CapeOX (oxaliplatin 130 mg/m^2^ on day 1 and capecitabine 1000 mg/m^2^ twice daily on days 1–14), both scheduled every 3 weeks until 6 months of perioperative chemotherapy was completed.

### 2.3. Follow-up

All patients were monitored to every week during treatment, and were regularly followed up after completion of the treatment once every 3 months during the first 2 years, and then every 6 months thereafter. The follow-up evaluation consists of patient medical history, physical examination, MRI examination for pelvic, chest CT, and carcinoembryonic antigen tests. Additionally, whole-body bone scan was performed when patient complaint about pain in bone. Endoscopy and imaging examination were performed according to clinical indications.

### 2.4. Statistical analysis

We aimed to report the pCR rate after neoadjuvant chemoradiotherapy and 3-year oncological outcomes for OS and DFS. In addition, we investigated the adverse factors related to the survival. Survival data and differences were calculated with the Kaplan–Meier method. OS was defined as the time of initial treatment to death from any reason or the last follow-up. DFS was defined as the time of initial treatment to the date of disease locoregional recurrence or distant metastasis or death. Patients who did not experience the interest events were analyzed as censored observations at the time of last follow-up. A Cox proportional hazards regression model was used to perform multivariate analysis of prognostic factors. A two-sided *P* value ≤ .05 was considered significant. Categorical variables between the arms were compared by the chi-square test. DFS and OS were determined using the Kaplan–Meier method, and survival curves were compared via the log-rank test. All statistical analyses were performed using SPSS 26.0 software (IBM Corporation, Amenk, New York, NY).

## 3. Results

### 3.1. Patient details

Between March 2014 and June 2020, a total of 139 consecutive local advanced rectal cancer patients were diagnosed and treated at our institution with nCRT and TME with/without postoperative chemotherapy. A total of 121 of 139 patients were eligible for this analysis, while the other 18 patients were excluded owing to ineligibility, among whom 10 were found to have distant metastases preoperatively or intraoperatively, 2 were complicated with other diseases and 6 were older than 75 years. Patients’ characteristics are listed in Table 1. Median patient age was 56-year-old (range, 22–75-year-old). Among the 121 patients, 24 achieved a pCR (19.8%). Potentially high-risk factors defined by imaging were as follows: 59 patients (48.8%) had a positive MRF status (by MRI); 42 patients (34.7%) were staged as cT4 disease with the invasion of adjacent organ(s); 48 patients (39.7%) had low-lying tumors; and 73 patients (60.3%) had cN2 disease (≥4 lymph nodes). Based on postoperative pathology, 15 patients had N1C disease (tumor deposits in the pelvic tissues); 20 patients had perineural invasion; pCR was obtained in 24 cases and 20, 39, and 38 patients had pathological stages I, II, and III, respectively. In the present study, patients were staged on the basis of the eighth edition American Joint Committee on Cancer/International Union for Cancer Control staging criteria.

**Table 1:** Baseline clinical characteristics of patient.

Clinical characteristics	Patients (n = 121)
Age, yr	
Median (range)	56 (22–75)
Sex, n (%)	
Male	76 (62.8)
Female	45(37.2)
Distance from the anal verge (cm), n (%)	
0–≤ 10 (lower-mid)	48 (39.7)
>5–12 (mid to high)	73 (60.3)
Clinical T stage, n (%)	
T3	79 (65.3)
T4	42 (34.7)
Clinical N stage, n (%)	
N0	12 (9.9)
N1	36 (29.8)
N2	73 (60.3)
MRI MRF status, n (%)	
Negative	62 (51.2)
Positive	59 (48.8)
PCR, n (%)	24 (19.8)
Pathological T stage	
T0	27 (22.3)
T1	2 (1.7)
T2	20 (16.5)
T3	65 (53.7)
T4	7 (5.8)
Pathological N stage	
N0	84(69.4)
N1	27 (22.3)
N2	10 (8.3)
Lymphatic vascular invasion	
Yes	2 (1.6)
No	119 (98.3)
Perineural invasion (PNI)	
Yes	15 (12.4)
No	106 (87.6)
TRG	
0	24 (19.8)
1	38 (31.4)
2	51 (42.1)
3	8 (6.6)
Pathological stage	
0	24 (19.8)
I	20 (16.5)
II	39 (32.2)
III	38 (31.4)

MRF = mesorectal fasciae, MRI = magnetic resonance imaging, pCR = pathologic complete response, TRG = tumor regression grade.

Only 2 patients failed to complete the planned course of nCRT. Both of them refused nCRT at 18 Gy and 20 Gy because of treatment toxicity. A total of 119 patients completed neoadjuvant radiotherapy, all these patients received concurrent chemotherapy, 75 with concurrent capecitabine (CapRT), 12 with concurrent capecitabine and irinotecan (CapIriRT) and 34 with concurrent capecitabine and oxaliplatin (CapeOXRT). According to our institutional protocol, 24 patients with the UGT1A1 genotype *1*1 or *1*28 were enrolled in the CinClare study^[15]^ and randomized to the experimental group (radiation with capecitabine combined with irinotecan followed by irinotecan and capecitabine) or the control group (radiation with concurrent capecitabine (12), followed by CapeOX). The median time from the end of chemoradiotherapy to surgery was 8 weeks (interquartile range: 1–20 weeks). During the interval between nCRT and surgery, 100 patients received chemotherapy, and 21 patients did not receive any consolidation therapy. 121 patients underwent TME surgery.

Seventy-six (62.8%) patients received adjuvant chemotherapy after TME. Thirty-seven (30.6%) patients did not undergo systemic adjuvant treatment: 11 due to a pCR, 10 without high-risk pathological factors, 2 due to patient refusal, 8 due to poor postoperative recovery or complications, 2 because of medical contraindications, and 4 due to rapid disease progression. In addition, 8 (6.6%) patients did not complete a full chemotherapy course due to therapeutic toxicity.

Two patients had a positive circumferential resection margin (CRM) status on pathological specimens. Lymphatic vascular invasion occurred in 3 patients. Twenty-four (19.8%) patients achieved a pathological complete response (pCR) after TME. Thirty-eight (31.4%) had only microscopic foci, 51 (42.1%) had partial regression, and 8 (6.6%) reported no tumor regression.

Twenty-seven of 121 patients who received TME have developed recurrent or metastatic cancer. Distant failure was the most common mode of failure and occurred in 25 patients. The most common organ of distant metastasis was the lung (n = 9). Other types of distant failure included liver disease (n = 3), bone metastasis (n = 3), peritoneal disease (n = 2), para-aortic lymph node metastasis (n = 1), inguinal lymph node metastasis (n = 1), and breast metastasis (n = 1). Simultaneous multiple sites metastases were detected in 5 individuals. Three patients were observed pelvic recurrence, and 1 patient presented with synchronous local and distant diseases. Most of the failures were asymptomatic and were detected by an increase in carcinoembryonic antigen levels followed by subsequent imaging. A total of 18 patients have died from tumor-related causes.

### 3.2. Survival outcomes

Survival curves were generated to evaluate OS and DFS by Kaplan–Meier method. A Cox proportional hazards regression model was used to performed multivariate analyses in order to find out different prognostic factors associated with survival (univariate analysis is shown in Table 2). Related factors for 3-year OS and DFS were investigated in univariate and multivariable analyses that included age, sex, MRF by MRI, tumor regression grade, perineural invasion, pathological T stage, pathological N stage, ypTNM stage, pathological N1c stage (tumor deposits), completion of adjuvant chemotherapy at a full course, pathological complete response, synchronous chemotherapy regimen, and chemotherapy during the interval before surgery.

**Table 2:** Univariate analysis of OS and DFS.

Factors	OS		DFS	
	Univariate χ^2^	*P* values	Univariate χ^2^	*P* values
Age	0.266	.606	1.829	.176
Sex (female vs male)	0.16	.693	0.407	.524
MRI positive MRF status (yes vs no)	9.968	.002	1.379	.240
Tumor regression grade (per 1 grade increase)	30.710	.000	14.280	.003
Perineural invasion (PNI)	9.191	.002	2.246	.134
Pathological T stage	4.606	.330	7.475	.113
Pathological N stage	23.621	.000	19.557	.000
Pathological stage (PCR, I, II, III, IV)	18.079	.000	21.206	.000
Pathological N1c stage (yes vs no)	13.684	.000	15.358	.000
Completion of adjuvant chemotherapy (yes vs no)	0.015	.902	1.697	.193
Pathological complete response (yes vs no)	2.978	.084	6.386	.012
Synchronous chemotherapy regimen	2.659	.265	0.915	.633
Chemotherapy during the interval before surgery (yes vs no)	0.121	.728	0.037	.847

DFS = disease-free survival, MRF = mesorectal fasciae, MRI = magnetic resonance imaging, OS = overall survival, pCR = pathologic complete response.

**Figure 1.**
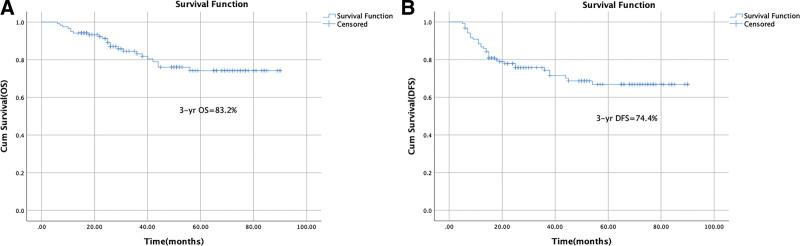
Kaplan–Meier curves. Kaplan–Meier estimates for (A) OS and (B) DFS (n = 121). DFS = disease-free survival, OS = overall survival.

**Figure 2.**
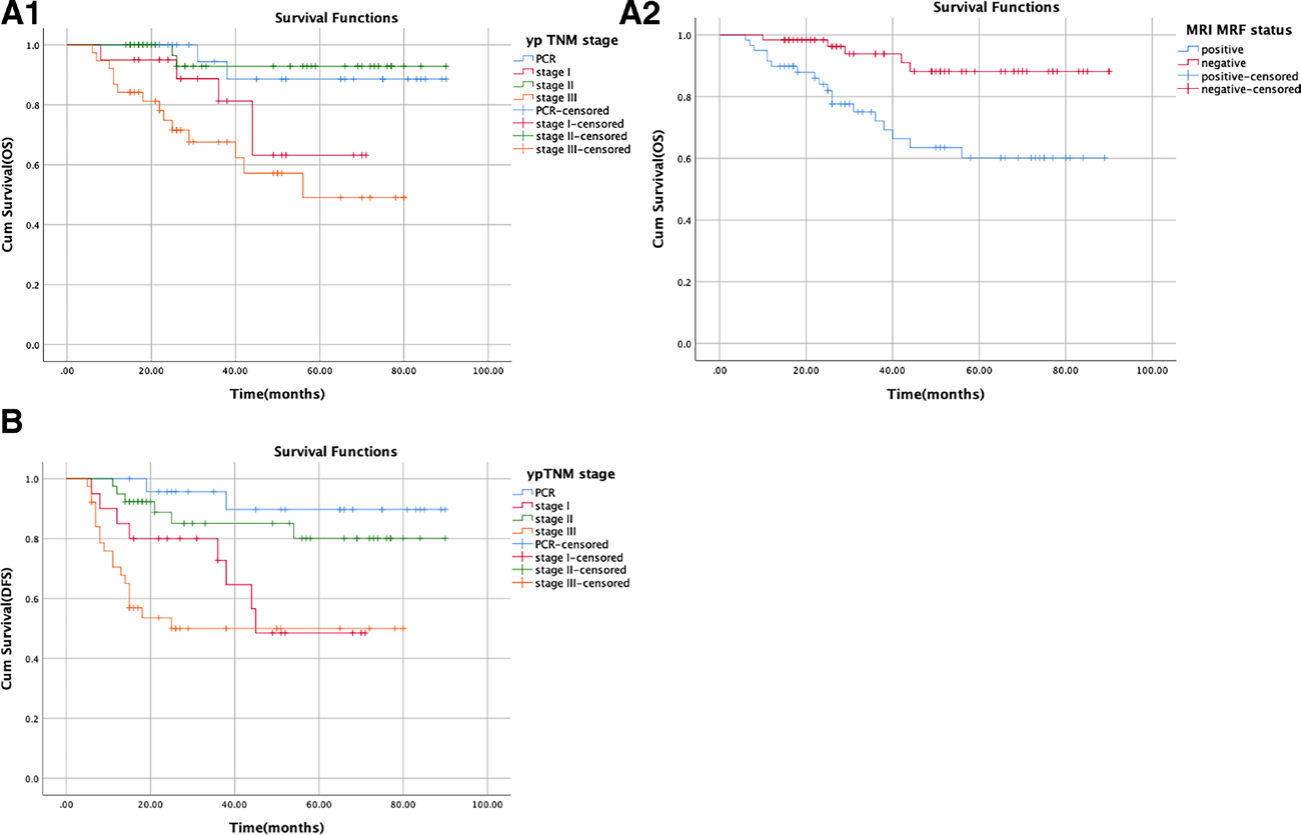
(A-1) Kaplan–Meier estimates for OS of subgroup (ypTNM stage). (A-2) Kaplan–Meier estimates for OS of subgroup (MRI MRF status). (B) Kaplan–Meier estimates for DFS of subgroup (ypTNM stage). Kaplan–Meier estimate for subgroups; DFS = disease-free survival, MRF = mesorectal fasciae, MRI = magnetic resonance imaging, OS = overall survival, pCR = pathologic complete response, ypTNM stage = TNM stage after neoadjuvant therapy.

The 3-year OS was 83.2% (Fig. 1A). The 3-year DFS was 74.4% (Fig. 1B). Univariate analysis of OS showed that MRI MRF status, tumor regression grade, pathological N stage, ypTNM stage, pathological N1c stage, and perineural invasion had *P* values ≤ .05. After multivariate adjustment, MRI MRF status (χ^2^ = 7.431, *P* = .006), and ypTNM stage (χ^2^ = 8.192, *P* = .042) remained significant (Fig. 2A1 and A2). Univariate analysis revealed that tumor regression grade, pathological N stage, ypTNM stage, pCR and pathological N1c stage (tumor deposits) potentially influenced DFS, but only ypTNM stage (χ^2^ = 14.101, *P* = .003) remained related to DFS after multivariate analysis (Fig. 2B).

## 4. Discussion

The pCR rate in the study was 19.8%, which is comparable to local and international clinical trial data. In the German CAO/ARO/AIO-04 trial,^[11]^ a pCR rate was 17% in the fluorouracil and oxaliplatin arm and 13% in the fluorouracil arm. The CAO/ARO/AIO-12 trial^[12]^ reported that a pCR of the intention-to-treat individuals was achieved in 17% of group A patients who were received 3 cycles induction chemotherapy of fluorouracil, leucovorin, and oxaliplatin before fluorouracil/oxaliplatin CRT (50.4 Gy) and in 25% of group B patients with consolidation chemotherapy after CRT. Other TNT-related studies^[18]^ had similar pCR rates. A Chinese study^[15]^ in which we included 24 patients had a pCR rate of up to 30% in the experimental group. It is worth mentioning that 5 (41.7%) out of 12 patients who received CapIriRT, 8 (23.5%) out of 34 in the CapeOXRT arm and 11 (14.7%) out of 75 in the CapRT arm achieved pCR in our institution, respectively. According to the UGT1A1 genotype, choosing radiotherapy in combination with irinotecan and capecitabine as neoadjuvant therapy may be an innovative, promising treatment option.^[15]^ It is worth putting forward that the addition of irinotecan was also associated with increase in the occurrence of grade 3 to 4 toxicities. Long-term follow-up will determine if the improvement in the pCR is translated into improved survival.

Thirty-four patients were treated with oxaliplatin combined with capecitabine chemotherapy during radiotherapy. Adding oxaliplatin to fluorouracil-based preoperative chemoradiotherapy and postoperative chemotherapy significantly improved the disease-free survival of patients with stage II/III rectal cancer compared with a former fluorouracil-based combined regimen (based on CAO/ARO/AIO-94).^[1]^ Seventy-five patients in our study received combinations with concurrent capecitabine chemotherapy during radiotherapy based on clinical studies.^[9,10]^ Twelve patients received concurrent capecitabine and irinotecan (CapIriRT),^[15]^ and the efficacy and safety were demonstrated in previous clinical studies.^[22,23]^ There were no obviously differences were found in OS and DFS among different concurrent chemotherapy regimens in our study. Some studies have shown that adding additional chemotherapy between the end of nCRT and surgery improved survival outcomes.^[12]^ After neoadjuvant therapy, if there was little tumor regression based on MRI, we gave the patient more interval consolidation therapy. Three to 4 cycles of chemotherapy were arranged for 7 patients. Generally, TME surgery was scheduled for 8 to 12 weeks after the completion of nCRT. We found that 45 patients failed to complete all of the chemotherapy cycles. There were also no significant differences in OS and DFS among patients who did and did not complete postoperative chemotherapy at the full dose (yes vs no) in our study. It is consistent with another study that showed the value of adjuvant chemotherapy might be controversial.^[24]^ However, Sandra-Petrescu et al^[25]^ pointed that the complete administration of chemotherapy cycles after surgery were associated with improved the overall and disease-free survival at 5 years in patients with locally advanced rectal cancer.

Pelvic MRI were required to performed for all our patients to help staging evaluation before radiation and surgery. In our study, the MRI MRF status was an independent prognostic factor for OS. Preoperative MRI examination can confirm the status of MRF, which can help to make more individualized treatment plans. During follow-up, regular MRI can detect local recurrence and metastasis earlier and help to provide timely intervention treatment.

The 3-year OS was 83.2%, and the 3-year DFS was 74.7% (Fig. 1). After multivariate adjustment, MRI MRF status (χ^2^ = 7.431, *P* = .006), and ypTNM stage (χ^2^ = 8.192, *P* = .042) remained significant for OS (Fig. 2A1-2). The 3-year OS with PCR, ypstage I, II, III was 94.4%, 92.9%, 81.3%, and 67.6%, respectively. The 3-year OS with or without positive MRF was 72.1% and 93.8%, respectively. YpTNM stage (χ^2^ = 14.101, *P* = .003) was the only independent risk factor for DFS after multivariate Cox regression analysis (Fig. 2B). The 3-year DFS rates with PCR, ypstage I, II, III were 95.7%, 85.1%, 72.7%, and 50%, respectively.

One study from Hong Kong^[13]^ reported that the 3-year and 5-year OS rates were 77.2% and 63.9%, respectively. The 3-year and 5-year DFS rates were 69.4% and 68.3%, respectively. The survival of patients with positive CRM was significantly reduced compared with those with negative CRM and the 3-year OS for CRM-negative patients was 88.3%. After multivariate adjustment, pathological positive CRM status and histological grade remained significant for OS. Pathological positive CRM status and pathological N stage remained significant for DFS. A phase III Germany study^[9]^ showed that the 5-year OS in the capecitabine arm was not inferior to that in the fluorouracil arm (76% vs 67%; *P* = .0004); 3-year DFS was 75% and 67% in the capecitabine group and the fluorouracil group (*P* = 0·07), respectively. The results of FFCD 9203^[3]^ showed that there was no difference for neoadjuvant radiotherapy with or without concurrent fluorouracil and leucovorin in T3-4 rectal cancers in the 5-year OS and 5-year PFS in both arms (67.9% vs 67.4%; and 55.5% vs 59.4%). The 5-year OS rate for all groups was 65.2% in the EORTC Radiotherapy Group Trial 22921.^[5]^ The German CAO/ARO/AIO-04 study^[26]^ published its final results in 2015: the 3-year DFS was 75.9% in the experimental group (infusion fluorouracil and oxaliplatin) and 71.2% in the control group in which patients were underwent chemotherapy with fluorouracil-based combined modality regimen. The 3-year disease-free survival was 73% in the German CAO/ARO/AIO-12 trial.^[27]^ Five-year disease-free survival was 80.8% (95% CI, 77.9–83.7) in the FOLFOX group and 78.6% (95% CI, 75.4–81.8) in the chemoradiotherapy group reported in a recent clinical trial.^[28]^

Several large trials of TNT^[12,17,29,30]^ have reported that TNT had the following therapeutic advantages: it significantly increased the completion of systemic treatment, improved tumor regression, reduced the incidence of tumor-related treatment metastasis and recurrence and increased the cCR ratio and W&W probability. Choosing the watch-and-wait strategy for carefully selected patients who have achieved a cCR with neoadjuvant therapy may be feasible.^[20]^ Immunotherapy has also shown some preliminary results in neoadjuvant treatment for locally advanced rectal cancer.^[31–34]^ As research progresses, individualized treatment plans need to be developed for specific patients.

The main reason for treatment failure in our group was distant metastasis, so ways to implement systemic treatment more effectively is important. An increasing number of clinical studies have shown that the initial treatment of TNT is satisfactory. At present, we are conducting research on TNT or on choosing a chemotherapy regimen according to the UGT1A1 genotype, aiming to obtain better clinical results and reduce distant failures. Encouraging oncological results were obtained at the 3-year follow-up but needed to be confirmed with longer follow-up. A more detailed analysis of the toxicity and quality of life will be published separately. There are some limitations in our study, such as insufficient sample size and short follow-up time. We will extend the duration of treatment and continue follow-up to overcome the associated deficiencies.

## 5. Conclusion

Patients with stage II/III rectal cancer achieved a relatively high pCR rate after neoadjuvant therapy, and the 3-year survival showed promising oncological outcomes. YpTNM stage was significantly associated with DFS; MRI MRF status and ypTNM stage were significant factors for OS after multivariate analysis. Distant metastasis is the dominant mode of treatment failure, and it is crucial to optimize systemic treatment for newly diagnosed patients.

## Author contributions

**Data curation:** Fei Li.

**Formal analysis:** Fei Li, Chi Zhang, Liping Xu.

**Writing – original draft:** Fei Li, Chi Zhang, Liping Xu, Dongsheng Zhang, Yan Leng, Chenjiang Wu.

**Writing – review & editing:** Sheng Zhang, Jiayan Chen, Xinchen Sun.
